# The Central Hospitals Board

**Published:** 1896-05-30

**Authors:** W. H. Allchin

**Affiliations:** Physician to the Westminster Hospital


### The Institutional Workshop.

### THE CENTRAL HOSPITALS BOARD.

By W. H. Allchin, M.D., F.R.C.P., Physician to the Westminster Hospital.

I trust that Mr. Holland will acquit me of intentional discourtesy in having neglected to reply last week to his comments on my former letter. I can only plead press of work as an excuse.

Let me say at once that I think those actually interested in hospital matters are much indebted to Mr. Holland for the sincerity and disinterested energy with which he has taken up one of the most difficult of social problems, and for the good work he has already accomplished, for which I fear he is likely to receive but scant gratitude. At the same time, this does not prevent me from holding quite a different view as to the desirability of a Central Hospital Board being formed to the one he advocates, though I cannot admit our differences are due to my being an "insider" and he an "outsider." Surely his six years of hospital management have made him as much an "insider" as I am, though I must confess to having been a member of a hospital committee for more than three times that period. No; I suspect the grounds which underlie our fundamental disagreement are to be found in the following remark: "I have found," says Mr. Holland, "that there is no focussed experience outside the pages of the 'Hospital Annual'; there is no centre of information; all the hospitals are at sixes and sevens on questions of vital importance; they overlap each other's work, there is no intercommunication of any sort, and if we want information and advice, it must be got at the expense of much time and trouble in going round all the hospitals, or in lengthy correspondence."

Now, anyone unacquainted with the actual facts of the case would conclude from this that the management of the London hospitals is a chaotic muddle, whereas, from what I know, they are regularly and efficiently and, in the main, economically administered, each one in its own way, and on its own lines, troubled I admit by the ever-pressing lack of funds. If it be the wish of Mr. Holland and those who think with him to have all the hospitals conducted on an identical plan, and uniform in all their details, then I admit a Central Board of some kind would be an essential. But I for one should regard that as a very undesirable purpose to accomplish, and one most difficult of attainment, with a strong probability of the hospitals themselves failing in their essential work, whilst it was being settled which was the best plan of management. And, moreover, a Central Board without legal powers would be quite unable to bring about such uniformity, and the possession of compulsory powers Mr. Holland particularly disclaims. To my mind the individuality of the hospitals is their special merit, and I can foresee all kinds of difficulties if that were lost. Again, my experience would not lead me to believe that there are so many burning questions which call for united action on the part of the hospitals such as to necessitate that close intercommunication which Mr. Holland would seem to desire. Now and then, once or twice perhaps in a year, it is convenient to know what is the practice of other institutions in respect to this or that question, but the information is readily and quickly obtained.

Perhaps it were better I should say I am only considering the question as it affects the large general hospitals. The special hospitals, with but few exceptions, I am not concerned with. Most of them in my opinion ought never to have been allowed to exist, and much of the present difficulty connected with hospital relief I regard as due to them. It is very possible that they might benefit much from the existence of a Central Hospital Board, especially if it had the power of the purse behind it, and still more if compulsory powers to enforce its directions were superadded. Besides this difference which exists between Mr. Holland and myself on the question of a need for a Central Board, we further disagree, as he very fairly points out, in our anticipations concerning such a Board were it established. Whilst to me the "possible evils" loom large, to him "certain good" predominates. On the plane of prophecy, it has been well said, we are all equal, unless, of course, the question is begged, which somehow seems to me to be the case here. Anyhow, I am prepared to enumerate the evils, and I should like to be a little more sure of the "certain good."

I cannot quite reconcile the following remarks of Mr. Holland which appear in contiguous columns, unless he has permitted his politeness to go further than he meant. "I put," he says, "the arguments against a Central Board with fairness and force," "but if anyone will re-read his (my) letter it will be seen there is no single argument brought forward against the Central Board principle, but only a repetition of evil prophecy and vague fear." It is more than possible that I did not express myself clearly; let me try again. My objections to a Central Board of any kind are very real, viz.:—

Because I regard the individuality and autonomy of the hospitals as essential, and a Central Board to be effective must interfere with that individuality and autonomy.

Because the work of "supervision" of the hospitals in London, to be effective, would be quite beyond the limits of a Board's capabilities, unless all were to be conducted on a uniform plan in all details.

Because the local and personal interest in hospitals would diminish in proportion as a Central Board came to usurp the functions of the present governing bodies of the hospitals.

Because I believe the notion that uniformity is a good thing in itself is a mistaken one, and that any such condition, if attained, would go far to destroy the benefits that follow the present friendly rivalry between similar institutions managed from within.

Because I believe that hospitals require to be let alone to work out their own improvement.

Holding such views I feel justified in styling the proposal to form a Central Board "impractical and mischievous."

Lastly, I cannot agree with Mr. Holland when he suggests that the ability of many of the members of the Lords' Committee would render it likely that they were right in recommending that a Central Hospital

Board should be formed, plausible as such a suggestion might be. It would rather appear to me that in the course of their inquiry their lordships found that they had entered on a subject more wide-searching and difficult than the chairman had any idea of when he moved for the committee; and that the proposal of a Central Board was an easy way at hand out of their difficulty.

I think this explanation is warranted when the way in which they shirked the important point of the out-patients department is remembered—a subject which the committee were well qualified to deal with in an authoritative manner. But whilst they neglected the main questions, they were pleased to be precise on the very technical point of what should or should not be the professional qualifications of the medical staffs of the general hospitals in London—a matter upon which their opinion was absolutely valueless.

It is for such reasons as these that the committee's report has never carried any weight with hospital authorities, who have fully recognised that a great opportunity was thrown away.

